# Trends in disease burden of type 2 diabetes, stroke, and hypertensive heart disease attributable to high BMI in China: 1990–2019

**DOI:** 10.1515/med-2024-1087

**Published:** 2024-11-29

**Authors:** Yunchao Wang, Junlin Jiang, Zhongxin Zhu

**Affiliations:** Department of General Medicine, The First People’s Hospital of Xiaoshan District, Xiaoshan Affiliated Hospital of Wenzhou Medical University, Hangzhou, Zhejiang, China; Clinical Research Center, The First People’s Hospital of Xiaoshan District, Xiaoshan Affiliated Hospital of Wenzhou Medical University, Hangzhou, Zhejiang, China

**Keywords:** disease burden, type 2 diabetes mellitus, stroke, hypertension, body mass index

## Abstract

**Background:**

High body mass index (BMI) is a significant risk factor for non-communicable diseases; however, its impact on disease burden in China remains understudied. This study aimed to analyze trends in the burden of type 2 diabetes mellitus (T2DM), stroke, and hypertensive heart disease (HHD) attributable to high BMI in China from 1990 to 2019.

**Methods:**

We utilized data from the Global Burden of Disease 2019 study, quantifying disease burden through years lived with disability (YLDs), years of life lost (YLLs), and disability-adjusted life years (DALYs). Joinpoint regression analysis was employed to determine temporal trends.

**Results:**

The study revealed distinct gender-specific temporal trends. Men exhibited a consistent increase in disease burden across all three conditions. Women showed more nuanced patterns: a gradual rise in T2DM burden, an inverted U-shaped trend for stroke, and a U-shaped trend for HHD in terms of age-standardized DALYs. Age-specific analysis demonstrated that the burden of T2DM and stroke peaked in the 70–74-year age group, whereas HHD-related DALYs continued to increase with advancing age.

**Conclusions:**

Our findings underscore the need for tailored obesity prevention and management strategies in Chinese healthcare settings, emphasizing early screening and intervention for high BMI, particularly in middle-aged and older adults.

## 1 Introduction

Elevated body mass index (BMI ≥ 25 kg/m²) is a complex risk factor with far-reaching public health implications [1]. In recent decades, obesity rates have surged globally, driven by a multifaceted interplay of excessive calorie consumption, sedentary lifestyles, and diverse socio-environmental and genetic influences [2]. This trend poses significant health risks, as obesity is a well-established contributor to numerous non-communicable diseases (NCDs), including type 2 diabetes (T2DM), hypertension, stroke, and cardiovascular disorders [3,4,5].

China has witnessed a dramatic surge in NCDs over recent decades, driven by an aging population and increased exposure to major risk factors [6]. The Global Burden of Disease (GBD) study identifies T2DM, stroke, and hypertensive heart disease (HHD) as primary contributors to the disease burden attributable to high BMI [7,8]. However, research specifically examining the BMI-attributable burden of these diseases in China remains scarce. This knowledge gap is particularly concerning given China’s rapid economic development and lifestyle changes, which have fueled a significant rise in obesity rates [9,10].

To address this critical knowledge gap, our study aims to elucidate the temporal trends in the burden of T2DM, stroke, and HHD attributable to high BMI in China from 1990 to 2019, leveraging data from the GBD 2019 study. This 30-year analysis provides a comprehensive picture of how high BMI’s health impacts have evolved in China. Our findings contribute to the existing literature on NCD burden in China and offer valuable evidence to inform public health strategies aimed at mitigating the impact of high BMI on population health.

## 2 Methods

### 2.1 Data source and study design

The data utilized in this study are available on the Global Health Data Exchange GBD Results Tool (http://ghdx.healthdata.org/gbd-2019). Building upon its predecessor, GBD 2017, the 2019 iteration incorporates additional data sources and refined estimation methodologies [11,12,13]. The GBD Study provides the detailed information regarding 367 causes of death and disability and 87 risk factors for 204 countries and territories [11]. GBD produced sound and up-to-date evidence of trends at the global, regional, and national levels as a result of the shift in the global agenda and increased focus on NCD and injury. GBD studies used three main standardized modeling tools to process data, model, and generate each estimation of disease by age, location, sex, and year-Cause of Death Ensemble, DisMod-MR, and Spatiotemporal Gaussian Process Regression [14].

Our investigation focuses specifically on the temporal trends of T2DM, stroke, and HHD attributable to high BMI in China. For adults (ages 20+), high BMI was defined as BMI ≥ 25 kg/m^2^ [12]. To quantify disease burden, we employed multiple metrics: years lived with disability (YLDs), years of life lost (YLLs), and disability-adjusted life years (DALYs). YLDs were calculated by multiplying the number of incident cases by the disability weight and mean disability duration. YLLs were derived by multiplying the estimated number of deaths by the standard life expectancy at the age of death. DALYs, representing the sum of YLDs and YLLs, provide a comprehensive measure of overall disease burden [15]. Our study is compliant with the Guidelines for Accurate and Transparent Health Estimates Reporting [16].

### 2.2 Statistical analysis

Joinpoint regression analysis was used to determine trends in YLDs, YLLs, and DALYs of T2DM, stroke, and HHD attributable to a high BMI. The maximum three joinpoints (four segments) were used to ensure the results were credible. For each trend segment identified by the mode, the annual percentage change (APC), calculated for each segmented line regression; average annual percent change (AAPC), calculated for the entire period; and 95% confidence interval (CI), were estimated [17]. The trend was considered decreasing (increasing) if APC and its 95% CI were both <0 (>0), and considered stable when the 95% CI overlapped with zero. Moreover, we analyzed the disease burden of different age groups in 2019. All analyses were performed using Joinpoint Regression Program (version 4.9.0.0, Statistical Methodology and Applications Branch, Surveillance Research Program, National Cancer Institute). *P* value <0.05 was considered significant.

## 3 Results

### 3.1 Disease burden of T2DM attributable to a high BMI

**Figure 1:**
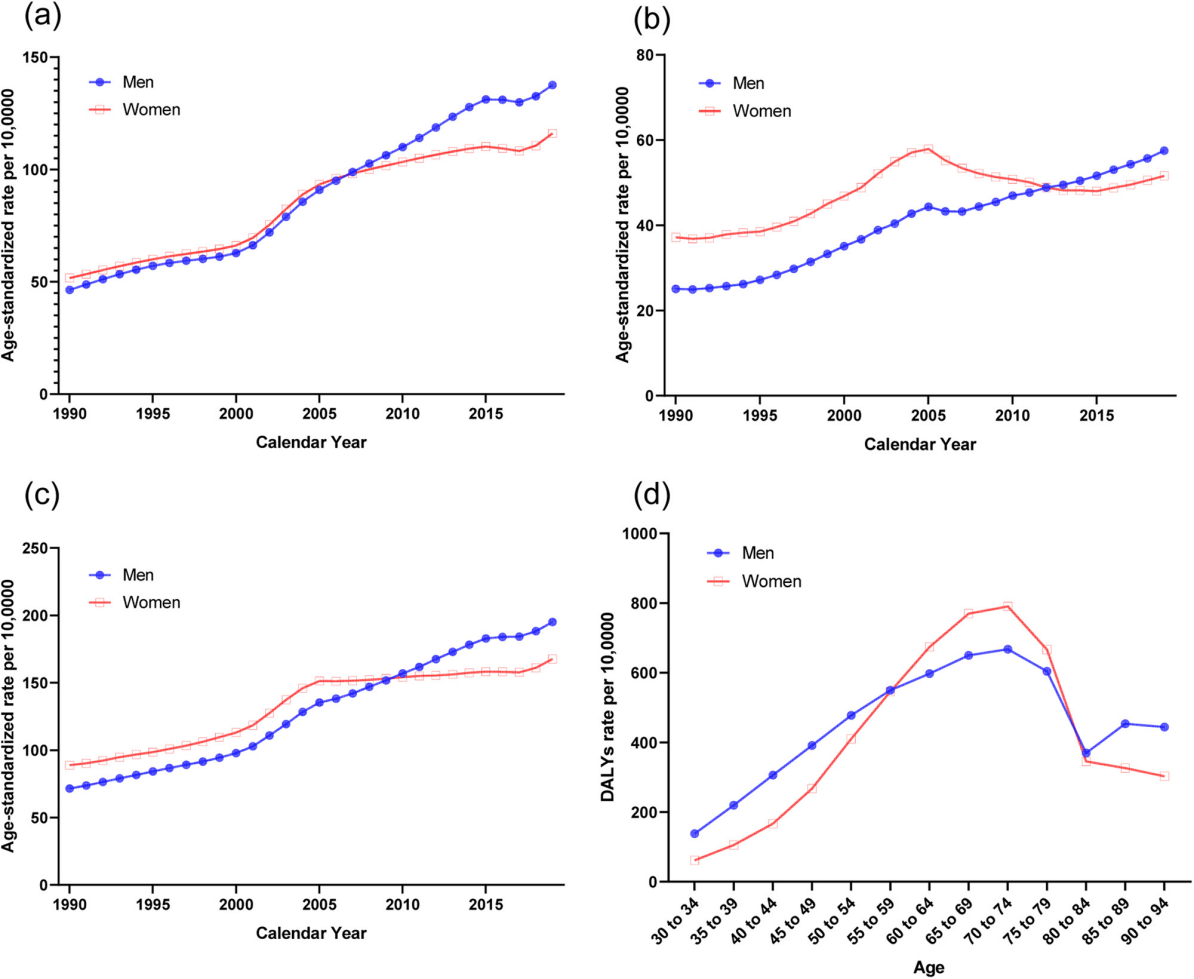
Burden of type 2 diabetes mellitus attributable to a high BMI by sex in China. (a) Age-standardized YLDs, rate per 100,000. (b) Age-standardized YLLs, rate per 100,000. (c) Age-standardized DALYs, rate per 100,000. (d) DALYs by age in 2019. Abbreviations: YLDs, years lived with disability; YLLs, years of life lost; DALYs, disability-adjusted life years.

The trends in the sex-specific age-standardized YLDs, YLLs, and DALYs of T2DM attributable to a high BMI in China from 1990 to 2019 are shown in Figure 1a–c, and the results of sex-specific Joinpoint regression analyses are shown in Table 1. Generally, the age-standardized YLDs, YLLs, and DALYs have increased steadily over the three decades for men (AAPC: YLDs, 3.6, 95% CI, 3.2, 4.1; YLLs, 2.9, 95% CI, 2.7, 3.1; DALYs, 3.5, 95% CI, 3.3, 3.7). For women, age-standardized YLDs, YLLs, and DALYs largely increased near the period 2000–2004, and then continuously increased for YLDs and DALYs. For YLLs, the trend decreased in the period 2004–2014 and again increased in recent years. The age-standardized DALYs rates due to a high BMI by age in 2019 are shown in Figure 1d. For the comparison from 30 to 34 years to 90 to 94 years, DALYs first increased and then decreased after age 70–74 years for both men and women.

**Table 1:** Results of the Joinpoint regression models for trend analysis of burdens of type 2 diabetes mellitus attributable to a high BMI in China from 1990 to 2019

Category	Sex	Year	APC	95% CI	AAPC (1990–2019)	95% CI
Type 2 diabetes mellitus						
YLDs	Males	1990–2001	3.0*	2.6, 3.3	3.6*	3.2, 4.1
		2001–2005	8.5*	5.8, 11.3		
		2005–2014	3.7*	3.2, 4.3		
		2014–2019	1.2*	0.0, 2.3		
	Females	1990–2001	2.5*	2.2, 2.8	2.7*	2.3, 3.1
		2001–2004	9.4*	5.4, 13.6		
		2004–2009	2.7*	1.5, 3.9		
		2009–2019	1.0*	0.7, 1.3		
YLLs	Males	1990–1994	0.9*	0.3, 1.5	2.9*	2.7, 3.1
		1994–2004	5.2*	5.0, 5.4		
		2004–2007	0.5	−1.4, 2.3		
		2007–2019	2.3*	2.2, 2.4		
	Females	1990–1996	1.0*	0.5, 1.5	1.2*	1.0, 1.4
		1996–2004	4.9*	4.5, 5.3		
		2004–2014	−1.9*	−2.2, −1.6		
		2014–2019	1.7*	1.0, 2.4		
DALYs	Males	1990–2001	3.2*	3.1, 3.4	3.5*	3.3, 3.7
		2001–2004	8.4*	6.4, 10.5		
		2004–2014	3.3*	3.1, 3.4		
		2014–2019	1.6*	1.1, 2.0		
	Females	1990–2000	2.3*	2.3, 2.4	2.2*	2.1, 2.3
		2000–2004	7.5*	6.9, 8.1		
		2004–2017	0.5*	0.5, 0.6		
		2017–2019	2.3*	1.2, 3.4		

^*^Significantly different from 0 (*P*  <  0.05). Abbreviations. YLDs: years lived with disability; YLLs: years of life lost; DALYs: disability-adjusted life years; APC: annual percent change; AAPC: average annual percent change; CI: confidence interval.

### 3.2 Disease burden of stroke attributable to a high BMI

**Figure 2:**
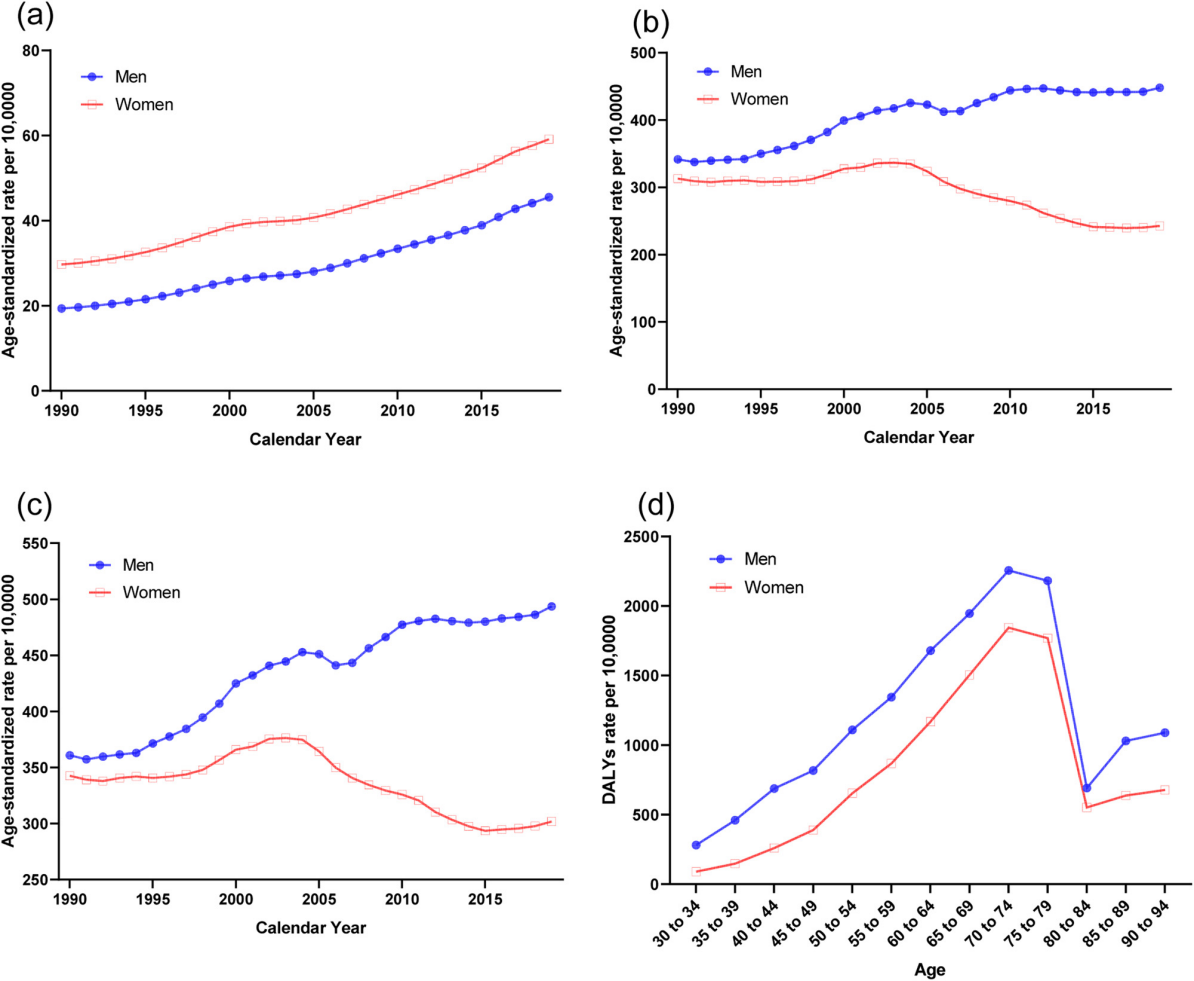
Burden of stroke attributable to a high BMI by sex in China. (a) Age-standardized YLDs, rate per 100,000. (b) Age-standardized YLLs, rate per 100,000. (c) Age-standardized DALYs, rate per 100,000. (d) DALYs by age in 2019. Abbreviations: YLDs, years lived with disability; YLLs, years of life lost; DALYs, disability-adjusted life years.

The trends in the sex-specific age-standardized YLDs, YLLs, and DALYs of stroke attributable to a high BMI in China from 1990 to 2019 are shown in Figure 2a–c, and the results of sex-specific Joinpoint regression analyses are shown in Table 2. The age-standardized YLDs, YLLs, and DALYs were higher in men than women. Generally, the age-standardized YLDs, YLLs, and DALYs have increased steadily over the three decades for men (AAPC: YLDs, 3.0, 95% CI, 2.8, 3.2; YLLs, 0.9, 95% CI, 0.7, 1.2; DALYs, 1.1, 95% CI, 0.8, 1.4). For women, there were a steady increasing trend for YLDs, a steady increasing trend from 1997 to 2003, and a largely decreasing from 2003 to 2015 for YLLs, and a steady increasing trend from 1999 to 2003, and a largely decreasing from 2003 to 2015 for DALYs. The age-standardized DALYs rates due to a high BMI by age in 2019 are shown in Figure 2d. For the comparison from 30 to 34 years to 90 to 94 years, DALYs first increased and then decreased after age 70–74 years for both men and women.

**Table 2:** Results of the Joinpoint regression models for trend analysis of burdens of stroke attributable to a high BMI in China from 1990 to 2019

Category	Sex	Year	APC	95% CI	AAPC (1990–2019)	95% CI
Stroke						
YLDs	Males	1990–1995	2.1*	1.7, 2.6	3.0*	2.8, 3.2
		1995–2000	3.9*	3.3, 4.5		
		2000–2005	1.5*	0.9, 2.1		
		2005–2019	3.5*	3.4, 3.6		
		1990–1994	1.6*	1.2, 2.0	2.4*	2.3, 2.5
		1994–2000	3.4*	3.1, 3.7		
		2000–2006	1.1*	0.8, 1.5		
		2006–2019	2.8*	2.7, 2.8		
YLLs	Males	1990–1995	0.4	−0.5, 1.2	0.9*	0.7, 1.2
		1995–2001	2.8*	1.9, 3.7		
		2001–2012	0.8*	0.5, 1.1		
		2012–2019	−0.0	−0.5, 0.5		
	Females	1990–1997	−0.1	−0.4, 0.2	−0.9*	−1.0, −0.7
		1997–2003	1.6*	1.1, 2.2		
		2003–2015	−2.9*	−3.0, −2.7		
		2015–2019	0.1	−0.6, 0.9		
DALYs	Males	1990–1995	0.5	−0.4, 1.3	1.1*	0.8, 1.4
		1995–2001	2.8*	2.0, 3.7		
		2001–2012	0.9*	0.6, 1.2		
		2012–2019	0.3	−0.2, 0.8		
	Females	1990–1996	0.0	−0.4, 0.4	−0.4*	−0.6, −0.3
		1996–2003	1.5*	1.1, 1.9		
		2003–2015	−2.2*	−2.3, −2.0		
		2015–2019	0.9*	0.1, 1.6		

^*^Significantly different from 0 (*P*  <  0.05). Abbreviations. YLDs: years lived with disability; YLLs: years of life lost; DALYs: disability-adjusted life years; APC: annual percent change; AAPC: average annual percent change; CI: confidence interval.

### 3.3 Disease burden of HHD attributable to a high BMI

**Figure 3:**
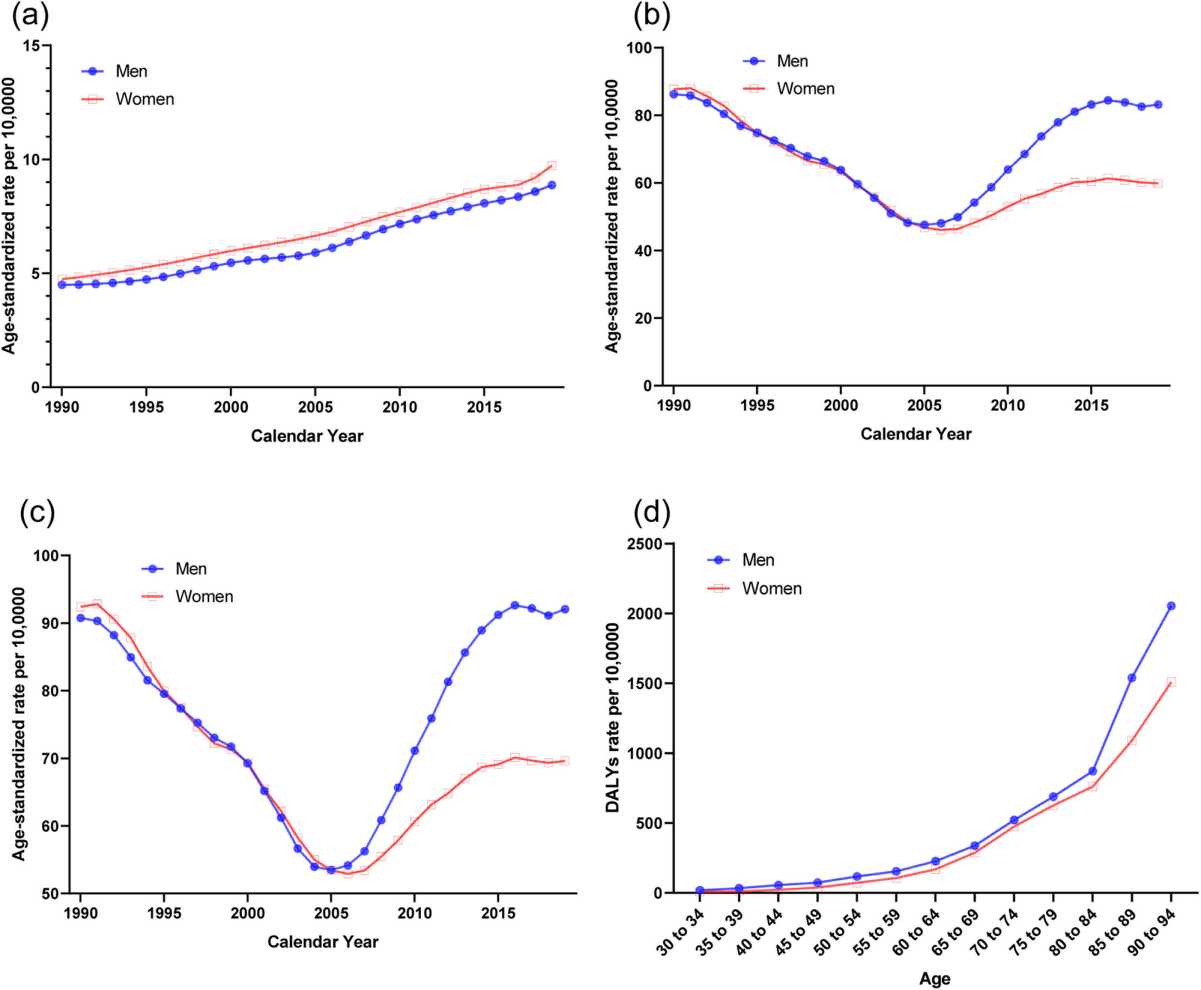
Burden of HHD attributable to a high BMI by sex in China. (a) Age-standardized YLDs, rate per 100,000. (b) Age-standardized YLLs, rate per 100,000. (c) Age-standardized DALYs, rate per 100,000. (d) DALYs by age in 2019. Abbreviations: YLDs, years lived with disability; YLLs, years of life lost; DALYs, disability-adjusted life years.

The trends in the sex-specific age-standardized YLDs, YLLs, and DALYs of HHD attributable to a high BMI in China from 1990 to 2019 are shown in Figure 3a–c, and the results of sex-specific Joinpoint regression analyses are shown in Table 3. For YLDs, there were steady increasing trends for both men and women from 1990 to 2019. For YLLs and DALYs, the change in trends was U-shaped for both men and women, with the turning point near 2005. The age-standardized DALYs rates due to a high BMI by age in 2019 are shown in Figure 2d. For the comparison from 30 to 34 years to 90 to 94 years, DALYs kept increasing with aging.

**Table 3:** Results of the Joinpoint regression models for trend analysis of burdens of HHD attributable to a high BMI in China from 1990 to 2019

Category	Sex	Year	APC	95% CI	AAPC (1990–2019)	95% CI
HHD						
YLDs	Males	1990–1993	0.6	−0.9, 2.1	2.4*	2.1, 2.6
		1993–2006	2.3*	2.1, 2.5		
		2006–2010	4.1*	2.6, 5.7		
		2010–2019	2.3*	2.0, 2.5		
	Females	1990–2005	2.3*	2.3, 2.4	2.5*	2.4, 2.7
		2005–2014	2.8*	2.7, 3.0		
		2014–2017	1.2	−0.0, 2.4		
		2017–2019	4.5*	3.3, 5.7		
YLLs	Males	1990–2000	−3.2*	−3.4, −2.9	−0.2	−0.4, 0.1
		2000–2005	−6.7*	−7.6, −5.7		
		2005–2014	7.0*	6.6, 7.4		
		2014–2019	0.1	−0.6, 0.8		
	Females	1990–2000	−3.6*	−3.9, −3.3	−1.4*	−1.7, −1.1
		2000–2006	−5.4*	−6.2, −4.6		
		2006–2014	4.0*	3.4, 4.5		
		2014–2019	−0.4	−1.2, 0.5		
DALYs	Males	1990–2000	−2.8*	−3.1, −2.6	0.0	−0.2, 0.2
		2000–2005	−5.9*	−6.7, −5.0		
		2005–2014	6.6*	6.3, 7.0		
		2014–2019	0.3	−0.4, 0.9		
	Females	1990–2000	−3.3*	−3.5, 3.0	−1.1*	−1.3, −0.8
		2000–2006	−4.6*	−5.3, −3.8		
		2006–2014	3.8*	3.3, 4.3		
		2014–2019	−0.0	−0.8, 0.8		

^*^Significantly different from 0 (*P*  <  0.05). Abbreviations. YLDs: years lived with disability; YLLs: years of life lost; DALYs: disability-adjusted life years; APC: annual percent change; AAPC: average annual percent change; CI: confidence interval.

## 4 Discussion

This study provides a comprehensive analysis of the disease burden attributable to high BMI for T2DM, stroke, and HHD in China from 1990 to 2019, utilizing Joinpoint regression analyses. Our findings reveal distinct gender-specific temporal trends: men exhibited a consistent increase in disease burden across all three conditions, while women showed more nuanced patterns. Specifically, for women, we observed a gradual rise in T2DM burden, an inverted U-shaped trend for stroke, and a U-shaped trend for HHD in terms of age-standardized DALYs. Moreover, the age-specific analysis demonstrated that the burden of T2DM and stroke peaked in the 70–74-year age group, whereas HHD-related DALYs continued to increase with advancing age.

These findings align with global health projections. The World Health Organization (WHO) estimates that the global prevalence of diabetes mellitus will increase from 9.3% to 10.2% by 2030 [18]. The significant rise in the prevalence of individuals with BMI ≥ 25 kg/m^2^ in both genders is particularly concerning, given the strong association between elevated BMI and T2DM risk [19]. Previous GBD studies have identified BMI as the leading risk factor for T2DM-related mortality [20]. Longitudinal data from the China Health and Nutrition Survey corroborate these findings, demonstrating that substantial long-term BMI gain significantly increases T2DM risk among Chinese adults [21]. The intricate interplay between obesity and T2DM pathophysiology underscores the potential for synergistic therapeutic approaches [22].

Stroke remains a significant global health challenge, ranking as the third-leading cause of DALYs and the second-leading cause of death worldwide in 2019 [23]. GBD studies have highlighted the substantial potential for reducing stroke burden through mitigation of modifiable risk factors [24]. Consequently, WHO recommendations for effective stroke prevention strategies encompass interventions targeting hypertension, dyslipidemia, unhealthy diet, physical inactivity, and high BMI [25]. Notably, high BMI emerged as the second leading risk factor for stroke in 2019 and exhibited the fastest growth rate among stroke risk factors between 1990 and 2019 [23]. Meta-analyses have further substantiated the independent association between high BMI and progressively increasing stroke risk [26]. However, the “Obesity Paradox” theory, suggesting potentially better outcomes for patients with high BMI compared to leaner or malnourished individuals [27], underscores the need for further high-quality research to elucidate the complex relationship between obesity and stroke outcomes.

Hypertension, a leading risk factor for cardiovascular disease, contributes significantly to the rapidly increasing disease burden in China [28]. The likelihood of developing chronic hypertension and cardiovascular events rises with increased visceral adiposity [29]. Obesity-induced alterations in nocturnal blood pressure patterns and the potential for blood pressure reduction through weight loss highlight the intricate relationship between adiposity and cardiovascular health [30]. The pathophysiological mechanisms underlying obesity-related HHD are multifaceted, involving pro-inflammatory states, increased sympathetic tone, and enhanced reactive oxygen species production [29]. The complex interplay between HHD and obesity, influenced by myriad contributing factors, emphasizes the critical importance of prevention and early intervention strategies for both conditions.

To our knowledge, this study represents the first investigation of the burden of T2DM, stroke, and HHD attributable to high BMI in China, utilizing the latest GBD study estimates. However, several limitations warrant consideration. First, the GBD study uses complex statistical models to estimate disease burden across different countries and time periods. While these models are sophisticated and aim to provide the best possible estimates, they are still based on available data and certain assumptions. This modeling process can introduce uncertainties and potential biases, especially in areas where primary data might be limited or of varying quality. Second, Asian populations tend to have a higher percentage of body fat and increased health risks at lower BMI levels compared to Western populations, and The World Health Organization and several Asian countries have suggested lower BMI cut-offs for overweight and obesity in Asian populations. Thus, by using the standard ≥25 kg/m^2^ definition, the GBD study might underestimate the true burden of disease attributable to high BMI in China [31,32]. Finally, the absence of urban–rural stratification in our analysis, due to data limitations, highlights the importance of considering regional variations in epidemiological studies, especially in a country as large and diverse as China [33].

In conclusion, our findings underscore the need for tailored obesity prevention and management strategies in Chinese healthcare settings, emphasizing early screening and intervention for high BMI, particularly in middle-aged and older adults, and integrating BMI management into existing chronic disease prevention programs to address the increasing burden of high BMI-related diseases in China. Our findings highlight the urgent need for comprehensive public health policies that address the economic impact of increasing obesity-related disease burden, promote healthy diets and physical activity, and tackle the social determinants of obesity to mitigate healthcare costs, productivity losses, and overall societal impact in China.
